# Recommendations for the Empirical Treatment of Complicated Urinary Tract Infections Using Surveillance Data on Antimicrobial Resistance in the Netherlands

**DOI:** 10.1371/journal.pone.0086634

**Published:** 2014-01-28

**Authors:** Maike Koningstein, Akke K. van der Bij, Marlieke E. A. de Kraker, Jos C. Monen, Jan Muilwijk, Sabine C. de Greeff, Suzanne E. Geerlings, Maurine A. Leverstein- van Hall, on behalf of the ISIS-AR Study Group

**Affiliations:** 1 Centre for Infectious Disease Control, National Institute for Public Health and the Environment (RIVM), Bilthoven, The Netherlands; 2 Department of Medical Microbiology, Reinier de Graaf Groep, Delft, The Netherlands; 3 Department of Internal Medicine, Division of Infectious Diseases, Centre for Infection and Immunity Amsterdam, Academic Medical Centre, Amsterdam, The Netherlands; 4 Department of Medical Microbiology and Infection Prevention, Bronovo Hospital, The Hague, the Netherlands; California Department of Public Health, United States of America

## Abstract

**Background:**

Complicated urinary tract infections (c-UTIs) are among the most common nosocomial infections and a substantial part of the antimicrobial agents used in hospitals is for the treatment of c-UTIs. Data from surveillance can be used to guide the empirical treatment choices of clinicians when treating c-UTIs. We therefore used nation-wide surveillance data to evaluate antimicrobial coverage of agents for the treatment of c-UTI in the Netherlands.

**Methods:**

We included the first isolate per patient of urine samples of hospitalised patients collected by the Infectious Disease Surveillance Information System for Antibiotic Resistance (ISIS-AR) in 2012, and determined the probability of inadequate coverage for antimicrobial agents based on species distribution and susceptibility. Analyses were repeated for various patient groups and hospital settings.

**Results:**

The most prevalent bacteria in 27,922 isolates of 23,357 patients were *Escherichia coli* (47%), *Enterococcus* spp. (14%), *Proteus mirabilis* (8%), and *Klebsiella pneumoniae* (7%). For all species combined, the probability of inadequate coverage was <5% for amoxicillin or amoxicillin-clavulanic acid combined with gentamicin and the carbapenems. When including gram-negative bacteria only, the probability of inadequate coverage was 4.0%, 2.7%, 2.3% and 1.7%, respectively, for amoxicillin, amoxicillin-clavulanic acid, a second or a third generation cephalosporin in combination with gentamicin, and the carbapenems (0.4%). There were only small variations in results among different patient groups and hospital settings.

**Conclusions:**

When excluding *Enterococcus* spp., considered as less virulent, and the carbapenems, considered as last-resort drugs, empirical treatment for c-UTI with the best chance of adequate coverage are one of the studied beta-lactam-gentamicin combinations. This study demonstrates the applicability of routine surveillance data for up-to-date clinical practice guidelines on empirical antimicrobial therapy, essential in patient care given the evolving bacterial susceptibility.

## Introduction

Urinary tract infections (UTIs) are among the most common nosocomial infections and a substantial part of the antimicrobial agents used in hospitals is for the treatment of UTIs [1]. Nosocomial UTIs are usually considered complicated infections since hospitalised patients with an UTI have a higher chance of sepsis and treatment-failure than patients attending a general practitioner [2], [3].

Another important predictor of treatment failure is antimicrobial resistance [4], [5], highlighting the importance of adequate recommendations for empirical treatment that are updated regularly given the evolving epidemiology and changing bacterial susceptibility [6], [7]. The Dutch Working Party on Antibiotic Policy (Stichting Werkgroep Antibioticabeleid [SWAB]) develops evidence-based guidelines for antimicrobial use in Dutch hospitals (www.swab.nl). The previous SWAB guideline for the antimicrobial treatment of complicated UTI (c-UTI) dates from 2006 [3]. Since then, resistance rates to amoxicillin-clavulanic acid, ciprofloxacin and the cephalosporins, which are recommended as empirical treatment in this guideline, have increased world-wide [8], [9], resulting in an update of the guideline in 2013 [10].

Data from surveillance can be used to guide the empirical treatment choices of clinicians [6], and to support treatment guidelines. Guidelines on antimicrobial use attempt to prevent discrepancies between empirical treatment and causative pathogens by recommending the least broad-spectrum agent with adequate bacterial coverage. Therefore, guidelines also play an important role in antibiotic stewardship by minimizing the use of broad-spectrum antimicrobial agents and subsequent ecological adverse effects of antimicrobial therapy, such as the selection of drug-resistant organisms [7].

*Escherichia coli* is the most common causative pathogen in UTIs worldwide [11]. However, data about the prevalence and distribution of other pathogens in UTIs are rare. Additionally, the prevalence of antimicrobial resistance differs greatly per country [12]–[14]. In this study, we used data from the Dutch Infectious Disease Surveillance Information System on Antimicrobial Resistance (ISIS-AR) to determine species distribution and antimicrobial susceptibility of urine isolates of hospitalised patients, and evaluated antimicrobial coverage of agents recommended for the empirical treatment of c-UTI in several patient groups and settings in the Netherlands.

## Materials and Methods

### Setting

ISIS-AR collects interpretations of antimicrobial susceptibility (i.e. susceptible, intermediate resistant and resistant), including underlying MIC values and disk zone diameters (if available), and patient data (i.e. age, gender, sample site, patient setting, department and date of admission in case of hospitalisation) of all routinely cultured bacterial species of participating medical microbiology laboratories located in various regions of the Netherlands [15]. In 2012, 32 laboratories (i.e. 65% of laboratories in the Netherlands) participated in ISIS-AR. These laboratories serve tertiary referral centres, teaching- and community hospitals, outpatient clinics, long-term care facilities, and general practitioners. Over 50% of the Dutch population is covered by ISIS-AR and its antimicrobial susceptibility data are considered representative for the Netherlands.

### Definition of c-UTI

ISIS-AR lacks clinical data and only collects antimicrobial susceptibility data of bacterial isolates with limited patient background data. We therefore defined a c-UTI as a positive urine sample (i.e., cultured uropathogen irrespective of the value of colony forming units (CFU)/ml since ISIS-AR does not collect information on CFU/ml) from a hospitalised patient since uncomplicated infections are rare in the hospital setting [2], [10], [16]. Due to the lack of patient data, we were not able to distinguish asymptomatic bacteruria (ASB) from a clinical UTI. However, it is not recommend to screen for ASB in the Netherlands [10]. We defined a c-UTI as hospital-associated if the urine sample was collected after the second day of hospital admission, otherwise the c-UTI was considered community-onset.

### Isolate selection

We included the first isolate per patient of urine samples of patients aged > = 18 years hospitalised from January to December 2012. Isolates from patients admitted at Intensive Care Units (ICU) were excluded since ICU-patients are usually more ill, receive more antimicrobials and often have urinary catheters. Additionally, rates of antimicrobial resistance at ICUs are higher than at non-ICU hospital departments [17], [18]. Urine samples from which only coagulase negative staphylococci (CoNS) were cultured (n = 428) or from which more than two pathogens were isolated were considered to represent contamination (n = 1530) and no infection [19].

### Antimicrobial susceptibility and antimicrobial coverage

For Enterobacteriaceae and other gram-negative bacteria, we reinterpreted the available MIC values of isolates for amoxicillin, amoxicillin- clavulanic acid, piperacillin-tazobactam, cefuroxime, ceftazidime, cefotaxime/ceftriaxone, ciprofloxacin, imipenem, meropenem, trimethoprim-sulphamethoxazole, nitrofurantoin and gentamicin as susceptible or non-susceptible using the European Committee for Antimicrobial Susceptibility Testing (EUCAST) 2012 (version 2.0) guidelines (www.eucast.org). For gram-positive bacteria, MIC values were unavailable for the majority of isolates. We therefore used antimicrobial susceptibility interpretations as reported by the participating laboratories. For the most common uropathogens, we determined to proportion of isolates non-susceptible for each antimicrobial agent separately, for the third generation cephalosporins as a group (3GC; non-susceptible to either ceftazidime, cefotaxime or ceftriaxone), for the carbapenems as a group (CARB; non-susceptible to either meropenem or imipenem), and for some specific antimicrobial combinations used for empirical treatment (i.e., amoxicillin, amoxicillin-clavulanic acid, cefuroxime or 3GC combined with gentamicin; non-susceptible to both agents).

To assess the probability of inadequate antimicrobial coverage, we calculated a weighted average of non-susceptibility for all uropathogens combined and for the gram-negative bacteria only. A weighted average implies that the distribution of species in urine samples was taken into account. For example, of the 14,022 *E. coli*, 45.6% is resistant to agent A (0.456*14,022), resulting in 6,394 resistant *E. coli*); of the 2,361 *Proteus mirabilis*, 22.9% is resistant to agent A (0.229*2,361) resulting in 541 resistant *P. mirabilis*. The weighted average of resistance of these two pathogens to agent A would be (14,022+2,361 = 16,383, of which 6,394+541 = 6,935 isolates are resistant) 6,935/16,383 = 42.3%, implying that in 42.3% of patients, the antimicrobial coverage of agent A is expected to be inadequate. When assessing the probability of inadequate coverage for a specific antimicrobial agent, we adjusted for pathogens that are considered intrinsically resistant to that agent (e.g., these pathogens were considered as resistant), according to the EUCAST expert rules [20].

### Statistical analysis

Proportions of non-susceptibility and probability of inadequate coverage were calculated as described as above. Fleiss Quadratic Approximation was used for the calculation of 95% confidence intervals (95% CI). To assess the generalizability of or our results we performed similar analyses for various patient groups and urine sample types, namely 1) community-onset versus hospital-associated c-UTI; 2) spontaneously passed midstream urine versus urine originating from catheters; 3) male patients versus female patients; 4) urine samples from all patients versus urine samples from patients with a blood sample with an identical species submitted within 7 days of the urine sample with that species (i.e., c-UTI versus urosepsis [median time between urine and blood sample collection: 0 days, mean time: 0.17 days and standard deviation 0.74 days]).

Finally, similar analyses were performed for different hospital settings, namely 1) community hospitals; 2) teaching hospitals; 3) university hospitals/tertiary referral centres. All three settings have their own specific patient population with university hospitals usually having the highest rates of antimicrobial resistance due to the more complicated nature of its patients. To assess regional differences, hospital settings were defined for each participating laboratory and analysis were repeated for each participating laboratory separately.

All data analyses were performed using SAS/STAT software, SAS System for Windows 9.3, SAS Institute Inc., Cary, NC, USA.

### Ethics statement

The data of the bacterial isolates and their susceptibility results used in this study belong to the microbiological laboratories participating in ISIS-AR and was obtained as part of routine clinical care in the past. Written or verbal consent of patients was therefore not obtained. Furthermore, all patient identifiers had been previously removed and data were analysed anonymously. According to the Dutch Medical Research Involving Human Subjects Act (WMO) this study was considered exempt from review by an Institutional Review Board.

## Results

### Pathogen distribution

We included 27,922 isolates from 23,357 patients. The most predominantly found pathogens were: *E. coli* (13,178; 47.2%), *Enterococcus* spp. (4,206; 15.1%), *P. mirabilis* (2,113; 7.6%), *Klebsiella. pneumoniae* (1,869; 6.7%), *Pseudomonas aeruginosa* (1,400; 5.0%), β-haemolytic streptococci group B (813; 2.9%), *Staphylococcus aureus* (751; 2.7%), *K. oxytoca* (607; 2.2%), *Enterobacter cloacae* (588; 2.1%), and *Morganella morganii* (284; 1.0%). The remaining 2.113 (7.6%) isolates were uncommon pathogens each accounting for less than 1% of the total number of isolates.

**Figure 1:**
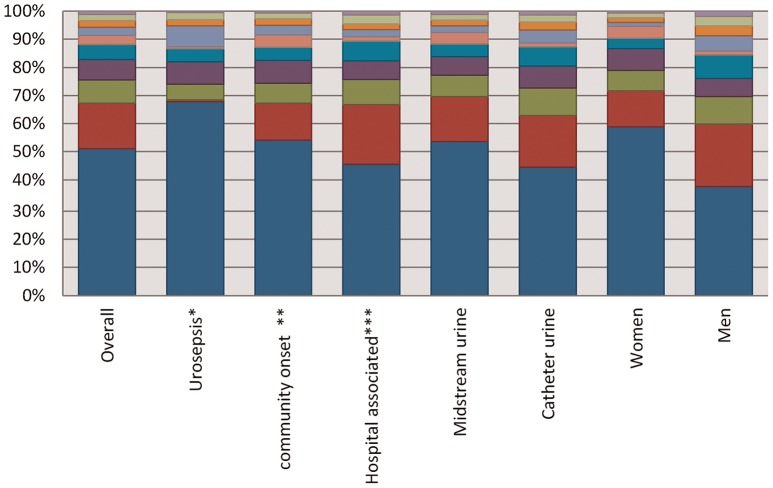
Pathogen distribution of various urine sample types and patient groups, ISIS-AR, the Netherlands, 2012. *We considered an infection to represent urosepsis when a blood specimen was submitted from the same patient, with the same pathogen within 7 days of a urinary specimen with that pathogen **We considered a UTI community onset if the urine sample was collected within two days after hospital admission *** We considered a UTI hospital associated if the urine sample was collected after the second day of hospital admission.

Figure 1 shows the distribution of pathogens per type of urine sample and various patient groups. *E. coli* were less frequently identified in hospital-associated c-UTI than in community-onset c-UTI (45.4%; 95% confidence interval [CI] 44.2%–46.7% versus 54.1%; 95%CI 53.0%–55.2%), while Enterococci, *P. mirabilis, P. aeruginosa*, and *E.cloacae* were more frequently found in hospital-associated c-UTI than in community-onset c-UTI. In urine samples from patients representing urosepsis (i.e., a positive blood sample with the same species), the top three pathogens was substantially different from samples of patients considered to have a c-UTI without a bloodstream infection. In samples from patients with urosepsis, *E. coli* was far more frequently isolated (67.8%; 95%CI 65.5%–70.0% versus 51.1%; 95%CI 50.5%–51.7%), while *Enterococcus* spp. were only isolated in 0.8% (95%CI 0.4–1.3%) versus 16.3% (95%CI 15.8–16.8%) urine samples.

### Antimicrobial coverage

Table 1a shows the percentage of non-susceptibility and probability of inadequate coverage for each antimicrobial agent of the most common pathogens isolated from urine samples. Table 1b shows the overall probability of inadequate coverage for gram-negative bacteria only. For all species combined, including both gram-negative and gram-positive pathogens, the probability of inadequate coverage was less than 5% for gentamicin, amoxicillin or amoxicillin-clavulanic acid combined with gentamicin and the carbapenems (Table 1a). When focussing on gram-negative bacteria, the probability of inadequate coverage of a second or a third generation cephalosporin in combination with gentamicin was less than 5% as well. A high probability of inadequate coverage was found for antimicrobial agents for which many of the pathogens are intrinsically resistant, such as amoxicillin or amoxicillin-clavulanic acid and cefuroxime. Also for trimethoprim-sulphamethoxazole there was a high probability of inadequate coverage. For ciprofloxacin, the probability of inadequate coverage was 11% (table 1a) and 14.8% (table 1b).

**Table 1:** The percentage of non-susceptibility (NS) for the most commonly isolated uropathogens separately, and the probability of inadequate coverage (e.g., weighted average) for uropathogens combined, ISIS-AR, the Netherlands 2012.

Table 1a. All uropathogens	AMX	AMC	CXM	CAZ	CTX/CRO	3GC	CARB	CIP	SXT	NIT#	GEN	AMO+GEN	AMC+ GEN	CXM+ GEN	3GC+GEN
	%NS	%NS	%NS	%NS	%NS	%NS	%NS	%NS	%NS	%NS	%NS	%NS	%NS	%NS	%NS
*E. coli*	46.8	18.1	12.6	4.9	5.8	6.3	0	17.2	28.4	2.5	6.0	5.0	2.8	2.4	1.6
*Enterococcus* spp.	16.7	16.7	IR	IR	IR	IR	24.4	NA	94.1	13.4	LLR	16.7	16.7	IR	IR
*P. mirabilis*?	22.2	10.7	1.3	1	0.9	1.3	0.1	10.6	30.4	IR	8.2	4.1	2.2	0.2	0.1
*K. pneumoniae*	IR	11.5	14.6	7.4	7.9	8.4	0.2	10.8	17.3	33.0	4.7	4.7	3.7	3.9	3.9
*P. aeruginosa*	IR	IR	IR	4	IR	IR	4.9	13.1	IR	IR	5.0	5.0	5.0	5.0	5.0
beta-haemolytic streptococci	0	0	0	NA	0.5	0.5	0	NA	18.3	3.6	NA	0	0	0	0
*S. aureus*	74.9	2.5	2.5	IR	2.5	IR	3.2	22.6	4.8	11	1.3	1.3	0.8	0.7	0.9
*K. oxytoca*	IR	11	13.2	1.7	2.8	3	0.2	4	4	5.1	1.3	1.3	1.9	1.2	0.7
*E. cloacae*	IR	IR	IR	13.5	26.2	26.9	0.7	8.9	10.6	21.8	7.9	7.9	7.9	7.9	7.0
*M. morganii*?	IR	IR	IR	15.3	20.4	22.1	0	13.8	17.7	70.6	8.6	8.6	8.6	8.6	2.2
Weighted average*	43.0	20.7	29.9	10.5	23.9	26.8	2.2	11.0	28.3	19.0	4.2	3.8	2.9	16.7	16.3
Table 1b. Gram-negative uropathogens only	AMX	AMC	CXM	CAZ	CTX/CRO	3GC	CARB	CIP	SXT	NIT#	GEN	AMO+GEN	AMC+GEN	CXM+GEN	3GC+GEN
Weighted average*	53.0	25.7	21.3	5.1	12.7	13.2	0.4	14.8	26.0	23.7	5.9	4.0	2.7	2.3	1.7

IR =  intrinsic resistant, NA = not available, LLR = low level resistance.

? We only included resistance to meropenem for this bacterium.

# For Enterobacteriaceae the breakpoint for *E. coli* was used.

* The distribution of species (e.g., number of isolates of each species) was taken into account when calculating the probability of inadequate coverage (e.g., weighted average); the overall resistance percentages are therefore the resistance percentages that would be encountered when choosing empirical treatment.

AMX: amoxicillin, AMC: amoxicillin-clavulanic acid, CXM: cefuroxime, CAZ: ceftazidime, CTX: cefotaxime, CRO: ceftriaxone, 3GC: 3rd generation cephalosporins, CARB: carbapenems, CIP: ciprofloxacin, SXT: trimethoprim-sulphamethoxazole, NIT: nitrofurantoin, GEN: gentamicin.

Since gram-negative bacteria represent the majority of causative pathogens, in particular in patients representing urosepsis (figure 1), we assessed the generalizability of our results for the gram-negative bacteria only, and found only minor variations in results among different patient groups and urinary sample types (table 2). In general, isolates from male samples were more resistant than isolates from female samples, with the largest differences in probability of inadequate coverage found for amoxicillin-clavulanic acid, cefuroxime, ceftazidime, third generation cephalosporins and ciprofloxacin. Resistance was lower in community-onset c-UTI than in hospital associated c-UTI, in particular for 3GC. However, for all patient groups and sample types the probability of inadequate coverage was below 5% for amoxicillin-clavulanic acid, cefuroxime, or a third generation cephalosporin in combination with gentamicin, and the carbapenems.

**Table 2:** The probability of inadequate coverage (e.g., weighted average) for gram-negative uropathogens (see table 1) in different groups of patients or urinary specimen types, ISIS-AR, the Netherlands 2012.

	AMX	AMC	CXM	CAZ	CTX/ CRO	3GC	CARB	CIP	SXT	NIT	GEN	AMO + GEN	AMC + GEN	CXM + GEN	3GC + GEN
All patients	53.0	25.7	21.3	5.1	12.7	13.2	0.4	14.8	26.0	23.7	5.9	4.0	2.7	2.3	1.7
Urosepsis*	53.4	19.7	14.4	7.2	12.4	9.1	0	20.6	33.9	2.6	7.1	6.3	3.4	3.4	2.4
Community onset**	51.1	18.7	13.4	4.7	5.4	5.7	0.2	15.9	25.0	19.1	5.8	4.4	3.0	2.5	1.6
Hospital associated***	52.4	22.8	16.8	6.7	7.8	8.2	0.7	13.2	24.5	25.0	6.8	5.3	3.4	3.1	2.5
Midstream urine	51.1	18.1	14.0	4.8	5.9	6.2	0.3	14.2	26.2	17.0	5.7	5.0	2.6	2.4	1.7
Catheter urine	54.8	20.0	15.5	5.7	6.6	6.9	0.6	16.6	27.5	23.0	5.8	4.9	3.0	2.6	2.0
Women	48.9	17.2	12.4	4.5	5.2	5.6	0.3	12.5	24.7	16.1	5.0	4.0	2.3	1.9	1.4
Men	59.0	25.2	19.2	6.7	8.6	8.5	0.7	19.6	28.7	26.2	8.0	6.6	4.5	3.8	2.8

* We considered an infection to represent urosepsis when a blood specimen was submitted from the same patient, with the same pathogen within 7 days of a urinary specimen with that pathogen.

** We considered a UTI community onset if the urine sample was collected within two days after hospital admission.

*** We considered a UTI hospital associated if the urine sample was collected after the second day of hospital admission.

AMX: amoxicillin, AMC: amoxicillin-clavulanic acid, CXM: cefuroxime, CAZ: ceftazidime, CTX: cefotaxime, CRO: ceftriaxone, 3GC: 3rd generation cephalosporins, CARB: carbapenems, CIP: ciprofloxacin, SXT: trimethoprim-sulphamethoxazole, NIT: nitrofurantoin, GEN: gentamicin.

There were variations in the probability of inadequate coverage between the different hospital settings (table 3) and different laboratories (data not shown) for gram-negative isolates. The probability of inadequate coverage for amoxicillin-clavulanic acid, third generation cephalosporins, ciprofloxacin, gentamicin, and combinations of amoxicillin-clavulanic acid and third generation cephalosporins with gentamicin were higher in university hospitals. Inadequate coverage for almost all hospital settings and laboratories was below 5% for amoxicillin-clavulanic acid, cefuroxime, and third generation cephalosporins in combination with gentamicin, and the carbapenems.

**Table 3:** The average probability of inadequate coverage, including range, for gram-negative uropathogens (see table 1) in different hospital settings*, ISIS-AR, the Netherlands 2012.

Hospital setting	Community		Teaching		University	
	Average (%)	Range (%)	Average (%)	Range (%)	Average (%)	Range (%)
AMX	51.8	50.7–53.0	51.8	50.6–52.9	54.3	51.7–56.9
AMC	18.9	18.1–19.8	18.7	17.9–19.5	31.7	29.4–34.2
CXM	14.1	13.4–14.9	15.0	14.2–15.7	15.2	13.4–17.2
3GC	6.3	5.9–6.9	6.5	6.0–7.0	8.9	7.5–10.4
CARB	0.4	0.2–0.5	0.5	0.4–0.7	0.3	0.1–0.7
CIP	14.8	14.1–15.6	14.9	14.2–15.6	16.0	14.3–18.0
SXT	25.7	24.8–26.7	25.7	24.7–26.6	27.8	25.6–30.2
NIT	20.4	19.6–21.3	19.6	18.7–20.4	13.8	12.1–15.7
GEN	5.5	5.0–6.0	5.9	5.4–6.4	9.9	8.5–11.5
AMX+GEN	4.7	4.2–5.2	5.3	4.8–5.8	3.6	2.7–4.7
AMC+GEN	2.6	2.3–2.9	3.0	2.6–3.4	5.3	4.3–6.6
CXM+GEN	2.3	2.0–2.6	2.6	2.3–3.0	3.3	2.5–4.4
3GC+GEN	1.6	1.3–1.9	1.9	1.6–2.2	2.9	2.1–3.9

* There were no outliers among individual centers. The average number of patients was 261 for community hospitals (median 257, range 59–616), 523 for teaching hospitals (median 501, range 172–1020) and 692 for university hospitals (median 692, range 665–719).

AMX: amoxicillin, AMC: amoxicillin-clavulanic acid, CXM: cefuroxime, 3GC: 3rd generation cephalosporins, CARB: carbapenems, CIP: ciprofloxacin, SXT: trimethoprim-sulphamethoxazole, NIT: nitrofurantoin, GEN: gentamicin.

## Discussion

This study provides current information regarding the distribution of pathogens and their antimicrobial susceptibility patterns in urine samples from hospitalised patients in the Netherlands. Furthermore, we show that routinely collected surveillance data on antimicrobial resistance are useful for developing guidelines on antimicrobial therapy.

The large amount of patient data in our study, enabling sub-analyses for different patients groups, distinguishes this study from previous studies on UTI in the Netherlands that found *E. coli* in 72% of urine samples of female general practice patients [11], or international studies that focussed on one specific patient group, such as patients with urinary catheters, ICU patients or outpatients only [21]–[23]. Therefore, this study provides additional information on resistance in UTI than is currently available. Studies that have the power to assess results for different patient groups are especially beneficially for the development of a national guideline since they provide information on the generalizability of data, but also on specific patient groups that might need tailored recommendations. We found some variations in the distribution of pathogens between different patients groups and urine sample types. For instance, in the majority of the urine samples from patients representing urosepsis, gram-negative bacteria were the most commonly isolated pathogens, while enterococci, which are the second most commonly isolated pathogens when including samples from all hospitalised patients, were hardly identified, suggesting that the coverage of enterococci in empirical therapy is questionable due to their low prevalence in severe c-UTI, such as urosepsis [24]. The probability of inadequate coverage also showed some small variations between different patient groups and hospital settings and resistance was lowest in community-onset infections and in infections among female patients, potentially affecting antimicrobial therapy choices when considering single agents. For example, resistance to ciprofloxacin is lower among female patients than male patients and resistance to 3GC as a single treatment agent is lower among community-onset c-UTI than hospital-associated c-UTI due to the lower prevalence of *P. aeruginosa* and *Enterobacter* spp. However, when considering a percentage of 5% as the upper limit for inadequate coverage that is often used for decision making on empiric therapy for life-threatening infections [25], there were no substantial variations among suitable agents. Differences in the probability of inadequate coverage for the cephalosporins when considering all uropathogens combined versus gram-negative uropathogens only were mainly contributed to the *Enterococcus* spp since they are intrinsically resistant. This resulted in a higher probability of inadequate coverage with cefuroxime or 3GC in combination with gentamicin when compared to amoxicillin-clavulanic acid in combination with gentamicin. However, when only gram-negative uropahogens are considered there are no clinically relevant differences in probability of inadequate coverage for amoxicillin-clavulanic acid in combination with gentamicin and the cephalosporins in combination with gentamicin.

Although our study benefits from the large amount of data, it is limited by the lack of clinical information on actual infections and patient treatment. However, we performed sub analysis on data of patients with a blood sample and a simultaneous urine sample both with identical species that are likely to represent life-threatening infections with the urinary tract as the source of infection. Results from these sub analysis identified *E. coli* as the major pathogen and susceptibility patterns found in this group of patients identify the same antimicrobial agents with a probability of inadequate coverage below 5% as found in other patient groups. Additionally, it is not routine practice to collect urine samples for microbiological testing in the case of asymptomatic bacteriuria in the Netherlands [10], suggesting that urine samples are collected only when infection is suspected. Our results show higher percentages of resistance for ciprofloxacin and lower percentages of resistance to gentamicin than reported in previous Dutch studies on antimicrobial resistance [26], [27], which might be explained by the specimen selection and the use of non-susceptibility instead of resistance.

When considering routine surveillance data of urine samples and when excluding *Enterococcus* spp., that have a low prevalence in serious c-UTI such as urosepsis, the most suitable empirical treatment for c-UTI in hospitalized patients should be intravenous therapy with amoxicillin, depending on the local resistance patterns with clavulanic acid, or a second or third generation cephalosporin, all combined with an aminoglycoside that have a useful additive role in the treatment of serious infections by gram-negative bacteria, such as c-UTI [28], [29]. In many settings a third generation cephalosporin without an aminoglycoside might be a good alternative, depending on the local resistance data and severity of patient symptoms or in case of community-onset c-UTI. Mono-therapy with a second generation cephalosporin seems no suitable option due to the high resistance in most patient groups and hospital settings. Fluoroquinolones are also no suitable first-line choice for empiric therapy but might be an option for oral therapy in non-hospitalized patients and less severely ill patients. After initial empiric therapy, definite antimicrobial therapy should be directed based on the available antimicrobial susceptibility test results.

Although we found differences in the pathogen distribution and antimicrobial coverage between men and women, we do not recommend separate guidelines for the empirical treatment of c-UTI in these two patient groups since no major differences were found among those antimicrobial agents with a probability of inadequate treatment below 10%. Additionally, we do not recommend last-resort agents, such as the carbapenems for empirical treatment since broad-spectrum agents are associated with the selection of drug-resistant organisms [7], [26].

The results from this study are in line with the recommendations of the recently revised SWAB guideline for empirical treatment of c-UTI [10], demonstrating the applicability of routine surveillance data in guideline development and that the regular analyses of data on resistance allows for the timely adaptation of guidelines on empirical antimicrobial therapy.
