# Correction: Recommendations for the Empirical Treatment of Complicated Urinary Tract Infections Using Surveillance Data on Antimicrobial Resistance in the Netherlands

**DOI:** 10.1371/journal.pone.0107785

**Published:** 2014-09-08

**Authors:** The PLOS ONE Staff

The formatting of the last Author’s name is incorrect. The correct name is: Maurine A. Leverstein-van Hall.

## References

[1] Koningstein M, van der Bij AK, de Kraker MEA, Monen JC, Muilwijk J, et al (2014) Recommendations for the Empirical Treatment of Complicated Urinary Tract Infections Using Surveillance Data on Antimicrobial Resistance in the Netherlands. PLoS ONE 9(1): e86634 doi:10.1371/journal.pone.0086634 24489755 10.1371/journal.pone.0086634 PMC3904917

